# Hollow Carbon Microspheres/MnO_2_ Nanosheets Composites: Hydrothermal Synthesis and Electrochemical Behaviors

**DOI:** 10.1007/s40820-014-0019-z

**Published:** 2014-11-14

**Authors:** Hui-li Fan, Fen Ran, Xuan-xuan Zhang, Hai-ming Song, Xiao-qin Niu, Ling-bin Kong, Long Kang

**Affiliations:** 1 State Key Laboratory of Advanced Processing and Recycling of Non-ferrous Metals, Lanzhou University of Technology, Lanzhou, 730050 People’s Republic of China; 2 School of Materials Science and Engineering, Lanzhou University of Technology, Lanzhou, 730050 People’s Republic of China

**Keywords:** Manganese oxide, Hollow carbon microspheres, Composite electrode, Supercapacitor

## Abstract

This article reported the electrochemical behaviors of a novel hollow carbon microspheres/manganese dioxide nanosheets (micro-HC/nano-MnO_2_) composite prepared by an in situ self-limiting deposition method under hydrothermal condition. The results of scanning electron microscopy reveal that MnO_2_ nanosheets homogeneously grow onto the surface of micro-HC to form a loose-packed microstructure. The quantity of MnO_2_ required in the electrode layer has thereby been reduced significantly, and higher specific capacitances have been achieved. The micro-HC/nano-MnO_2_ electrode presents a high capacitance of 239.0 F g^−1^ at a current density of 5 mA cm^−2^, which is a strong promise for high-rate electrochemical capacitive energy storage applications.

## Introduction

With the rapid depletion of fossil fuels and increasingly worsened environmental pollution caused by vast fossil fuel consumption, there is currently a strong demand to make efficient use of energy and to seek renewable and clean energy sources [1]. Supercapacitor is an attractive power source, which has properties intermediate to those of batteries and electrostatic capacitors [2–5]. Supercapacitors are capable of storing electrical charge but are distinguished from electrochemical cells as they are essentially maintenance-free, possess a longer life cycle, require a very simple charging circuit, experience no memory effect, and are generally much safer [6, 7]. Unfortunately, supercapacitors deliver an unsatisfactory energy density, which is lower than that of batteries [8]. Thus, most current research works have focused on energy density enhancement of supercapacitors. It is well-known that their performance depends intimately on the properties of their active electrode materials [9]. In general, metal oxides/hydroxides and conducting polymers store charge in a faradic or redox-type process similar to batteries, which enables high energy density but is, in general, kinetically unfavorable.

Among all these materials, manganese oxide (MnO_2_) has been conceived as a promising supercapacitive material because of its low cost, low toxicity, and environmental safety, as well as high theoretical capacities [10–12]. However, the utilization efficiency of active materials is usually very low due to the fact that the pseudo-capacitive reaction of MnO_2_ is a surface reaction and that only the surface or a very thin surface layer of the oxide can participate in the pseudo-capacitive reaction [13]. So, the highly ordered metallic nanostructures should be one of interests for the development of supercapacitors’ electrode materials [14]. Thus, a composite electrode material architecture which incorporates nanoscopic MnO_2_ film on materials that have outstanding electrical conductivity, excellent mechanical flexibility, large specific surface area, and high thermal and chemical stability support [e.g., carbon nanofoams, templated mesoporous carbon, carbon nanotubes (CNTs)] is thought as ideal to optimize both the electrochemical performance and mass-loading of the ultrathin MnO_2_ [15–17]. So, the supports should have high high-surface-area, good electrical conductivity, and most importantly lighter. Thus, the utilization efficiency of active materials MnO_2_ can been improved and higher specific capacitances have been achieved.

We present an alternative route here to construct a novel composite of hollow core/shell electrode material for supercapacitors by incorporating MnO_2_ nanosheets (nano-MnO_2_) onto hollow carbon microspheres (micro-HC). Maintaining such hollow carbon, the utilization efficiency of active materials MnO_2_ will be improved. The core material is micro-HC, which has been prepared by simple, efficient, and economical synthetic technique. The results showed that the as-prepared micro-HC/nano-MnO_2_ composite material had a considerably high specific capacitance of 239.0 F g^−1^ in neutral electrolytes at a current density of 5 mA cm^−2^. The effects of reaction temperature on structure and electrochemical performance of micro-HC/nano-MnO_2_ composite were also investigated.

## Experimental Sections

### Materials

Potassium persulfate (APS), potassium permanganate, methylacrylic acid (MAA), dimethylformamide (DMF), and absolute alcohol were purchased from Sinopharm Chemical Reagent Co. Ltd., and used as received without any further purification. Styrene (St) from Sinopharm Chemical Reagent Co. Ltd. was purified via distillation prior. Divinylbenzene (DVB) was purchased from J&K Scientific Ltd.

### Preparation of Crosslinked Poly (styrene-*co*-methylacrylic acid) Hollow Spheres Aggregations

Firstly, MAA (0.431 g) was dispersed in 100 mL deionized water, and followed by the addition of St dropwise in a single-necked round-bottom flask at room temperature. In this process, the mixture was stirred under a nitrogen atmosphere all the time. Sequentially, the temperature of the mixture was increased to 80 °C; APS (0.135 g) was added into the reaction system. The polymerization continued for 24 h at 80 °C. After the reaction, the non-crosslinked P(St-*co*-MAA) template spheres were centrifuged and washed with both ethanol and deionized water, and then dispersed in 150 mL deionized water. Secondly, the obtained non-crosslinked P(St-*co*-MAA) template spheres solution (100 mL), deionized water (55 mL), and APS (0.135 g) were mixed in a single-necked round-bottom flask at room temperature. The mixture was stirred under a nitrogen atmosphere for 30 min. The mixture of St (2.083 g), MAA (0.431 g), and DVB (0.078 g) was added into the reaction system drop by drop. The polymerization continued for 24 h at 80 °C. After the reaction, the prepared product was centrifuged and washed with both ethanol and deionized water, then dispersed in 150 mL DMF and stirred for 12 h. The crosslinked P(St-*co*-MAA) was centrifuged and washed with both ethanol and deionized water. In this process, P(St-*co*-MAA) template spheres were removed and finally, the crosslinked poly (styrene-methylacrylic acid) hollow spheres aggregations were obtained in a powder form.

### Preparation of Hollow Carbon Microspheres

Hollow carbon microspheres (micro-HC) were prepared through a simple polymer carbonization method involving two steps. The prepared crosslinked P(St-*co*-MAA) hollow spheres aggregations were firstly pre-oxidized at 320 °C for 5 h and subsequently pyrolyzed at 700 °C under nitrogen atmosphere for 2 h. After the above procedure, the obtained powder, called hollow carbon microspheres (micro-HC), was cooled down to room temperature [18]. The micro-HC was further modified using the nitric acid oxidation method. A typical process for modification of hollow carbon microspheres (micro-HC) can be summarized as follows: 0.1 g carbon powder was dispersed in 10 mL of nitric acid solution (65 wt%) and the reaction was kept at 80 °C under refluxing process for 2 h; after oxidation, samples were recovered and washed thoroughly with deionized water until the pH was close to 7; the resultant product was further dried at 60 °C for 16 h.

### Preparation of Micro-HC/nano-MnO_2_

In a typical synthesis of micro-HC/nano-MnO_2_ composites procedure, 100 mg HC was added into 300 mL deionized water and the solution was ultrasonically stirred for 1 h. 250 mg of KMnO_4_ was subsequently added to the solution, which was ultrasonically dispersed for 30 min. Then, the mixture was moved into a water bath with vigorous magnetic stirring. The morphology and nanostructure of HC/MnO_2_ composites can be tuned by adjusting solution temperature (30, 60, and 90 °C). The resulting samples were washed with deionized water several times then dried at 60 °C for 12 h.

### Structure Characterization

The microstructure and morphology of the as-prepared samples were characterized by transmission electron microscope (TEM, JEOL, JEM-2010, Japan) and field emission scanning electron microscope (SEM, JEOL, JSM-6701F, Japan). Crystallite structure was determined by X-ray diffraction (XRD) using a Rigaku D/MAX 2400 diffractometer (Japan) with CuKα radiation (*λ* = 1.5418 Å) operating at 40 kV and 60 mA. Thermo gravimetric analysis (TGA) and differential scanning calorimetry (DSC) were carried out in air at a heating rate of 10 °C min^−1^ on a NETZSCH STA 449F3.

### Electrode Preparation and Electrochemical Measurements

The working electrodes were prepared as follows. 80 wt% of micro-HC/nano-MnO_2_ was mixed with 7.5 wt% of acetylene black and 7.5 wt% of conducting graphite in an agate mortar until a homogeneous black powder was obtained. To this mixture, 5 wt% of poly (tetrafluoroethylene) was added together with a few drops of ethanol. The resulting paste was pressed at 10 MPa into nickel foam (ChangSha Lyrun New Material Co. Ltd., 90 PPI, 2 mm) then dried at 80 °C for 12 h. Each carbon electrode contained approximately 8 mg of electroactive material and had a geometric surface area ≈1 cm^2^.

A typical three-electrode glass cell equipped with a working electrode, a platinum foil counter electrode, and a saturated calomel reference electrode (SCE) was used for electrochemical measurements of the as-prepared working electrodes. All electrochemical measurements were performed using an electrochemical working station (CHI660C, Shanghai, China) in 1 M Na_2_SO_4_ aqueous solution at 25 °C. The corresponding specific capacitance was calculated from the following Eq. (1):

1 $$C_{m}=\frac{C}{m}=\frac{I\times \Delta t}{m\times \Delta V},$$

where *C*_*m*_ (F g^−1^) is the specific capacitance, *I*(A) is discharge current, ∆*t*(s) is the discharge time, ∆*V*(V) represents the potential drop during discharge process, and *m*(g) is the mass of the active material [19, 20].

## Results and Discussion

### Preparation and Characterization of Materials

**Scheme 1:**
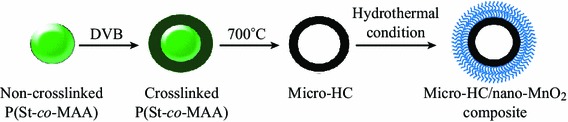
Illustration of the micro-HC/nano-MnO_2_ composite fabrication process

The detailed preparation process for the micro-HC/nano-MnO_2_ composite can be found in the experimental section and illustrated as Scheme 1. The method mainly involved the following procedures: (i) synthesis of non-crosslinked P(St-*co*-MAA) template spheres prepared by emulsion polymerization of St and MAA, which was further used as template spheres; (ii) addition of crosslinked polymer layer on the surface of the non-crosslinked P(St-*co*-MAA) template spheres to form a crosslinked/non-crosslinked core/shell microspheres; (iii) removing the non-crosslinked P(St-*co*-MAA) template spheres to get crosslinked hollow P(St-*co*-MAA) microspheres; (vi) carbonization of the crosslinked hollow P(St-*co*-MAA) microspheres via pre-oxidization and subsequently pyrolyzing process to obtain hollow carbon microspheres; (v) introducing MnO_2_ on surface of micro-HC by an in situ self-limiting deposition method under hydrothermal condition for the preparation of micro-HC/nano-MnO_2_ composite.

As shown by Kimet et al., carboxylated carbon can prevent metal oxide particles from agglomerating and contribute to the high dispersion of metal oxide nanoparticles by forming a bond between metal oxides and the surface carboxyl groups of the carbon [21], thus a simple modification of carbon was made using HNO_3_ as liquid oxidant. It is well-known that many oxygen-containing functional groups are introduced on the surface of the HNO_3_-modified carbon [22]. During the micro-HC/nano-MnO_2_ composite preparation process, the hollow carbon microspheres can firstly be completely dispersed in deionized water solution; then KMnO_4_ was added quickly with ultrasonic stirring for the in situ self-limiting deposition of nano-MnO_2_ on micro-HC surface. The reaction between carbon and KMnO_4_ can be described as the Eq. (2) [23].

2 $$4{\text{MnO}}_{4}^{-}+3\text{C}+{\text{H}}_{2}\text{O}\to 4{\text{MnO}}_{2}+{\text{CO}}_{3}^{2-}+2{\text{HCO}}_{3}^{-}$$

After the reaction between carbon and KMnO_4_, MnO_2_ nanosheets were formed on the carbon surface. Meanwhile, it should be noted that the carbon materials here were used as not only the template, but also the reducing agent, so it was consumed to a certain degree.

**Fig. 1:**
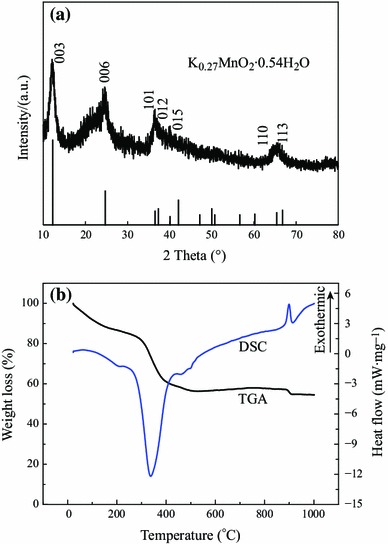
**a** XRD spectrum, and **b** TGA and DSC curves of micro-HC/nano-MnO_2_ composite

Figure 1a shows the XRD spectrum of the prepared micro-HC/nano-MnO_2_ composite. From the data in the figure, the structure of micro-HC/nano-MnO_2_ composite was proved to be of rhombohedral lamellar structure. Specifically, the four characteristic peaks of micro-HC/nano-MnO_2_ composite at 12°, 24°, 37°, and 66° can be indexed to K-birnessite-type MnO_2_ (JCPDS file no. 52-0556). The composition of this compound is K_0.27_MnO_2_(H_2_O)_0.54_. Birnessite is a type of two-dimensional layered structure manganese dioxide, which can facilitate cation intercalation/deintercalation with little structural rearrangement [24, 25]. In addition, the weak diffraction intensity of the samples reveals a poor crystallization or amorphous form of the MnO_2_-based materials prepared by facile chemical technique. This poor crystallization results in more transportation channels than a material with good crystallinity, and is favorable for the composite to exhibit a high capacitive performance [26].

The micro-HC/nano-MnO_2_ composite was also studied by TGA and DSC in the temperature range of 25–1,000 °C, as shown in Fig. 1b. TGA data showed the presence of three distinct stages: the sample weight decreasing gradually up to ~200 °C, a sharp reduction in sample weight below ~400 °C, and additional step in weight loss observed at ~900 °C. DSC data showed a broad exothermic peak at 340 °C and a narrow endothermic peak at 900 °C. The significant weight loss stage below ~200 °C was due to the loss of crystal water. In the temperature range of 200–400 °C, the weight loss can be attributed to the combustion of carbon. At temperatures exceeding ~400 °C the weight loss can be attributed to reduction of Mn^4+^ species and the formation of Mn_2_O_3_ [27, 28]. The weight loss at ~900 °C was related to the formation of Mn_3_O_4_ [28]. The TGA data showed that micro-HC/nano-MnO_2_ composite contained about 30 wt% of carbon and 60 wt% of MnO_2_. Hence, the hollow carbon microspheres may be totally coated by MnO_2_.

**Fig. 2:**
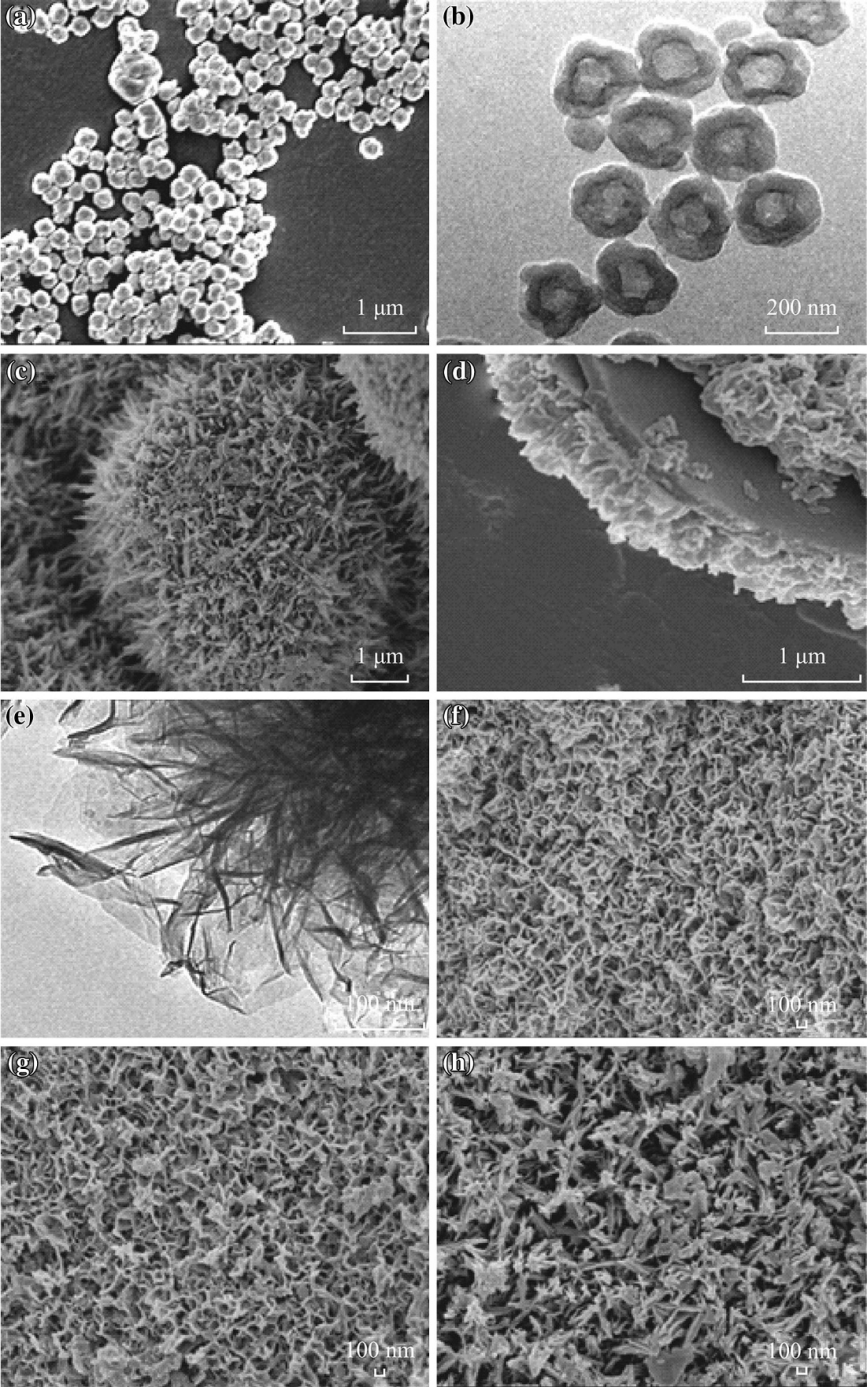
**a** SEM and **b** TEM images of micro-HC; **c**, **d** SEM and **e** TEM images of micro-HC/nano-MnO_2_ composite; **f**–**h** SEM images of micro-HC/nano-MnO_2_ composites prepared at different reaction temperature: **f** 30, **g** 60, and **h** 90 °C

The morphologies and microstructures of the micro-HC and the resulting micro-HC/nano-MnO_2_ composite were examined by SEM and TEM methods (see Fig. 2). Figure 2a–e depicts SEM and TEM images of the prepared micro-HC and micro-HC/nano-MnO_2_, revealing the hollow microstructure feature of carbon materials and the formation of loosely interconnected nanosheets packed in nanometer scale. The prepared micro-HC was evaluated to be 0.2–0.5 µm and the prepared micro-HC/nano-MnO_2_ composite had dandelion-like nanostructure bigger than the size of micro-HC. It was noteworthy that the dandelion-like nanostructure (which consists of interconnected nanosheets) showed anisotropic morphology characteristics, the formation of a loosely packed microstructure in the nanometer scale. In this view, the unique microstructures play a basic role in the electrochemical accessibility of electrolyte to MnO_2_ active material. Maintaining such hollow structures not only can increase the surface areas with much MnO_2_ nanosheets being incorporated on carbon materials, but also obtain the lighter electrode active material supports. Hence, the quantity of MnO_2_ required in the electrode layer has thereby been reduced significantly in favor of achieving higher specific capacitances.

The morphology of MnO_2_ with different dimensionalities and morphologies attract much attention due to their novel and unexpected properties [29], the reason of which is that the morphology of MnO_2_ closely relates to the specific surface area and therefore, the specific capacitance. In this work, the effects of reaction temperatures on the structure and properties of micro-HC/nano-MnO_2_ were studied. Figure 2f–h shows the different surface structures of nano-MnO_2_ fabricated at different temperatures. It can been seen from the pictures that the size of nanosheet is obtained, which is about 30 nm at 30 °C and 20 nm at 60 °C. With the reaction temperature increasing, the nanosheet became thinner. Even the thickness of each paper-shaped “petal” was about 10 nm and more nanosheets were curled into nano-rod at 90 °C. Compared with the petal-shaped MnO_2_, the specific surface area is larger, which is a good choice for electrochemical capacitors’ material [30]. As we well know that the unique microstructure played a basic role in electrochemical accessibility of electrolyte to MnO_2_ active material and the fast diffusion rate within the redox phase.

### Electrochemical Performance

**Fig. 3:**
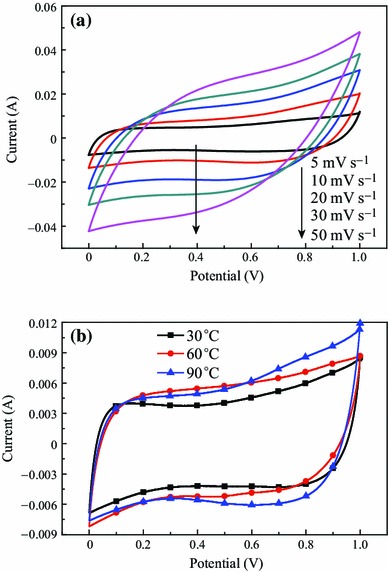
**a** Cyclic voltammograms of micro-HC/nano-MnO_2_ composite at different scanning rates; and **b** typical cyclic voltammograms of the micro-HC/nano-MnO_2_ composite prepared at different reaction temperature

Figure 3a shows the cyclic voltammetry (CV) curves (see) of the micro-HC/nano-MnO_2_ composite obtained at different scan rates. Despite the clear redox nature of the energy storage mechanism, MnOx-based electrodes can also demonstrate typical rectangular-shaped CV curves, analogous to non-faradaic energy storage mechanisms [30]. The CV curves are a good rectangle at the low scan rate. With the increase in scan rate, the CV curve shape changed to lancet from the rectangle shape; curve area increased and the symmetry of the CV curves became bad gradually. It can be indicated the reversibility of the electrode reaction was reduced.

Figure 3b shows the CVs of the micro-HC/nano-MnO_2_ composite prepared at different reaction temperatures. These curves show no peaks in the range between 0 and 1 V, making clear that the electrode capacitor was charged and discharged at a pseudoconstant rate over the complete voltammetric cycle [31]. At higher reaction temperature, the area of rectangular characteristic became larger, indicating almost ideal capacitive behavior for the obtained materials. The reason is that the petal-shaped MnO_2_, which may not be a good choice for electrochemical capacitors, could reduce the specific surface area, leading to capacitance fading.

**Fig. 4:**
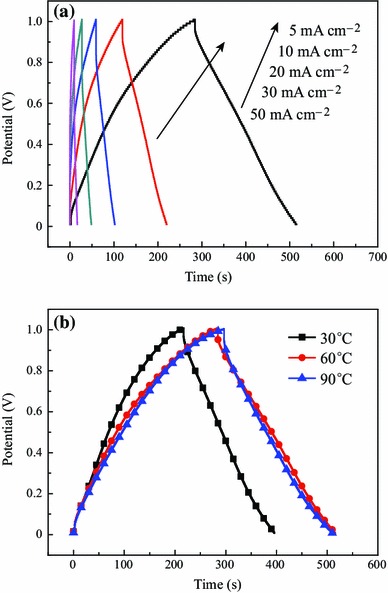
**a** Charge–discharge curves of micro-HC/nano-MnO_2_ composite at different current density; and **b** Charge–discharge curves of the micro-HC/nano-MnO_2_ composite prepared at different reaction temperature

The galvanostatic charge–discharge curves of micro-HC/nano-MnO_2_ composite were tested and shown in Fig. 4a. It can be seen that all the curves were highly linear and symmetrical at various current densities. This implied that the electrode had excellent electrochemical reversibility and charge–discharge properties. Moreover, the IR drops on all curves are similar and not obvious even at 50 mA cm^−2^, which indicated low overall resistance and excellent capacitive properties of this composite. At the low current density of 5 mA cm^−2^, the specific capacitance values of the micro-HC/nano-MnO_2_ composite calculated from the charge–discharge curves (see Fig. 4b) were 181.5, 229.0, and 239.0 F g^−1^ corresponding to the different reaction temperatures of 30, 60, and 90 °C, respectively. The energy storage characteristics of the composite were illustrated as follows: MnO_2_ sheets coating on the surfaces of micro-HC can pile up to form loosely interconnected nanosheets to improve the diffusion rate within the bulk of the prepared material during the charge–discharge process, which improves the electrochemical utilization of MnO_2_.

**Fig. 5:**
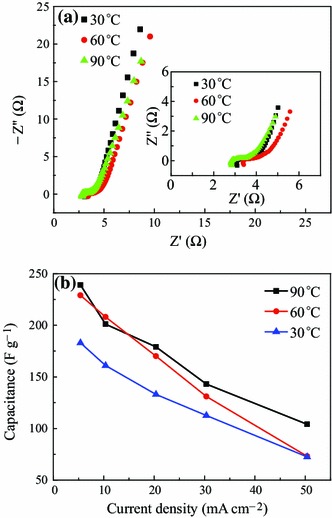
**a** Complex-plane impedance plot and the enlarged image of the high frequency region (*inset*); and **b** specific capacitance at different current densities of 5, 10, 20, 30, 40, and 50 mA cm^−2^ of micro-HC/nano-MnO_2_ composite prepared at different reaction temperature

To further investigate the capacitive behavior of micro-HC/nano-MnO_2_ composite, EIS test was also carried out over a frequency range from 10 kHz to 10 mHz (see Fig. 5a). It can be seen from the figure that a semicircle intersecting the real axis in the high frequency range is observed and then the plot transforms to a vertical line with decreasing frequency. In the range of high frequency, the point intersecting with the real axis reflects the internal resistance (*R*_*s*_) of the electrode material, including the total resistance of the ionic resistance of the electrolyte, the intrinsic resistance of active material, and the contact resistance at the active material/current collector interface. A semicircle reflects the electrochemical reaction impedance of the electrode, and a smaller semicircle means smaller charge transfer resistance (*R*_ct_). At the lower frequencies, a straight sloping line represents the diffusive resistance (Warburg’s impendence) of the electrolyte in electrode pores and the proton diffusion in host material [32]. The internal resistances (*R*_*b*_) of micro-HC/nano-MnO_2_ composite electrodes at 30, 60, and 90 °C, obtained from the intercept of the plots on real axis, are very similar (3.08, 3.40, and 2.80 Ω, respectively) [33]. However, the semicircle for the micro-HC/nano-MnO_2_ electrode was smaller, revealing a lower pseudo charge *R*_ct_ due to the good electrical conductivity. The reason is that the excellent interfacial contact between MnO_2_ and HC is of great benefit to fast transportation of electron throughout the whole electrode substrate. So, micro-HC in the composites not only acts as the skeleton for the deposition of MnO_2_ sheets but also the electronic conductive channels. At lower frequencies, each spectrum has high angles above 45°, corresponding to the lower Warburg impedance [34, 35], resulting from the effective nanoporous structure which facilitates the transport of electrolyte ions. Interestingly, the Warburg impedance at 90 °C electrode was the lowest, which was attributable to the effective porous structure of the nanosheets facilitating ionic transportation. To optimize the pore structures, the reaction temperature on specific capacitance of micro-HC/nano-MnO_2_ composite were systematically investigated (see Fig. 5b). The electrochemical capacitance performance of micro-HC/nano-MnO_2_ composite prepared at 90 °C was superior to that prepared at 30 and 60 °C. This indicated that when the temperature reached 90 °C, the effective interfacial area between MnO_2_ and the electrolyte, as well as the contact area between MnO_2_ and carbon resulted from the fair dispersion of nanoscale MnO_2_ on the increased conductive carbon. It can promote both the electrochemical utilization of MnO_2_ and the electrical conductivity of the electrode, the essential characters needed for pseudocapacitor with high power density.

**Fig. 6:**
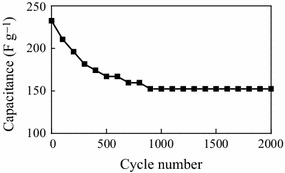
Cycle life of micro-HC/nano-MnO_2_ composite at a current density of 5 mA cm^−2^

To further understand the capacitive behavior of the micro-HC/nano-MnO_2_ composite, the cycling stability of the composite electrode was further examined by galvanostatic charge–discharge tests for 2,000 cycles at low current density of 5 mA cm^−2^ (cycle potential window: 0–1.0 V vs. SCE), as shown in Fig. 6. The specific capacitance gradually decreased with the increase of the cycle number, and 65.5 % of the initial specific capacitance remained after 2,000 cycles. However, the capacitance loss (34.5 %) occurred mainly during the first 1,000 cycles, which indicates that the Faraday reactions behave irreversibly or induce a degradation of the microstructure at the beginning of the cycle test. After that, the specific capacitance remained at 107 F g^−1^ until the 2,000 cycles showing a relatively good stability.

**Fig. 7:**
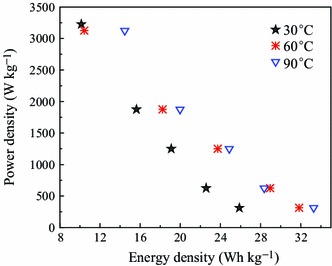
Ragone plots of micro-HC/nano-MnO_2_ composite

For further evaluation of the properties of the micro-HC/nano-MnO_2_ composite electrode, power density and energy density, are usually used to characterize the electrochemical performance of supercapacitors. The Ragone plot for the electrochemical micro-HC/nano-MnO_2_ composite in 1.0 mol L^−1^ Na_2_SO_4_ electrolyte is presented in Fig. 7. Due to the larger potential window and the higher specific capacitances, the application of micro-HC/nano-MnO_2_ composite as the positive electrode greatly improved the energy density of supercapacitors. Meanwhile, the energy density of the micro-HC/nano-MnO_2_ at 90 °C is much higher than that of micro-HC/nano-MnO_2_ at 30 and 60 °C, which means that the micro-HC/nano-MnO_2_ at 90 °C is superior in terms of both energy and power. The energy density attains 33.28 Wh kg^−1^ at a power density of 312.57 W kg^−1^, and still remains 14.47 Wh kg^−1^ at a power density of 3,124.80 W kg^−1^. The above results demonstrate that micro-HC/nano-MnO_2_ is very promising as an advanced electrode for supercapacitors.

## Conclusions

A novel method was used to synthesize micro-HC/nano-MnO_2_ composites through self-limiting deposition of nanoscale MnO_2_ on the surface of carbon under hydrothermal method. The composite with high-rate transportation of both electrolyte ions and electrons throughout the electrode matrix has superior electrochemical utilization of MnO_2_, resulting in the excellent electrochemical performance. The electrochemical studies indicated that the prepared micro-HC/nano-MnO_2_ composite presented high capacitance of 239.0 F g^−1^ in 1 M Na_2_SO_4_ solution. Through tuning the reaction temperature, the specific capacitance of micro-HC/nano-MnO_2_ composite prepared at 90 °C were higher than that of micro-HC/nano-MnO_2_ composite prepared at 30 and 60 °C. The facile synthesis approach may pave the way for successfully employing carbon-based composites for microelectronics, photovoltaic, chemical sensors and energy storage, and conversion applications.
